# A Comparison of the Mental Health Legislations in the Australian Capital Territory (ACT) and England and Wales (E&W) and the Resulting Impacts on Involuntary Hospitalisation Rates, Average Detention Lengths and Tribunal Reviews

**DOI:** 10.1192/bjo.2025.10234

**Published:** 2025-06-20

**Authors:** Anju Soni, Ian Treasaden

**Affiliations:** 1 Canberra Health Services, Canberra, Australia; 2 West London NHS Trust, London, United Kingdom

## Abstract

**Aims:** With reform planned for the England and Wales (E&W) Mental Health Act (MHA)1983 as amended by the MHA 2007, a comparison of the current E&W Act with the Australian Capital Territory (ACT) MHA 2015 was undertaken, including to examine whether differences were associated with variations in involuntary hospitalisation and Tribunal reviews.

**Methods:** A comparative analysis was conducted examining the E&W and ACT MHAs. Cross-jurisdictional analysis incorporated datasets from the Office for National Statistics (E&W) and ACT Health Services for three key metrics: per capita involuntary admission rates, mean duration of detentions, and frequency of Tribunal reviews, and analysed in relation to both legislative frameworks.

**Results:** The ACT mental disorder definition is narrower, includes intellectual disability but not personality disorder. Applications for the 3-month renewable ACT Psychiatric Treatment Order (PTO), limited to mental illness, are made to the ACT Civil and Administrative Tribunal (ACAT). It applies in hospital, the community, or correctional facilities. In E&W, the comparable Section 3: Admission for treatment detention period is 6 months. ACAT can authorise compulsory medication and ECT, a role in E&W of a Second Opinion Appointed Doctor (SOAD).

Criminal courts can request ACAT to assess a defendant for fitness to enter a plea and if not guilty by reason of mental impairment. This is the criminal courts’ role alone in E&W.

In E&W Crown Courts can make 'hybrid orders', a sentence of imprisonment allowing for treatment in hospital ensuring offenders are placed where their mental health needs can be best met.

Comparing E&W with ACT, the respective approximate populations served were 56 million and 431,000 and the annual involuntary hospitalisation numbers were 48,000–50,000 compared with 300–350 (statistically not significant; 0.087% compared with 0.075% of the population).

The average detention length was longer in E&W (30–45 days compared with 21–35) which has more Forensic units than the ACT.

Annual numbers of Tribunal reviews were 7000–8000 in E&W, while in ACT 200–250, per detained patient significantly more (p<0.0001).

**Conclusion:** The definition of mental disorder differs between the two legislations. The new MHA reforms in E&W will raise threshold for detention and require faster access to Tribunals. The ACT PTO applies to patients not only involuntarily hospitalised, but to those in the community and correctional facilities. This, with shorter detention periods and more frequent Tribunal reviews, may be factors in the shorter detention lengths in hospital in ACT compared with E&W.

Compared with E&W, there is a much greater role for the ACAT Mental Health Tribunal, including in authorising mental health orders, medication and ECT, which likely accounts for the significantly greater use of Tribunals in ACT.

In comparison to E&W, ACAT hearings are briefer and rely more on written than oral evidence. Innovatively, ACAT can be delegated by a Criminal Court to determine medico-legal issues in defendants.

